# Introduction of methicillin-resistant *Staphylococcus aureus* (MRSA) nasal polymerase chain reaction testing combined with pharmacist ordering and intervention reduces anti-MRSA antibiotic use in a multi-hospital system

**DOI:** 10.1017/ash.2025.10270

**Published:** 2026-01-14

**Authors:** Curtis D. Collins, Chiraag Gupta, Jennifer Chou, James Shen, Holly Murphy

**Affiliations:** 1 Trinity Health Ann Arbor, Ann Arbor, MI, USA; 2 Trinity Health Livonia, Livonia, MI, USA; 3 Trinity Health Oakland, Pontiac, MI, USA

## Abstract

Methicillin-resistant *Staphylococcus aureus* (MRSA) nasal polymerase chain reaction implementation combined with pharmacist oversight across four hospitals resulted in a 20.2% reduction in anti-MRSA agent standardized antimicrobial administration ratios with significant reductions across 17 of 23 patient care units, further supporting this approach as an effective, multi-center, antimicrobial stewardship strategy.

## Introduction

Inappropriate prescribing of vancomycin remains a significant challenge; over 27% of cases lack clear clinical justification.^1,2^ Methicillin-resistant *Staphylococcus aureus* (MRSA) nasal polymerase chain reaction (PCR) testing demonstrates a remarkably high negative predictive value in patients with pneumonia.^3^ Pharmacist-driven MRSA nasal PCR pneumonia protocols have proven effective, significantly reducing vancomycin use without compromising patient outcomes.^2,4–6^ Despite these advances, opportunities remain for further optimization, particularly in large, multi-center health-systems.

In early 2023, four community teaching hospitals within a Michigan health-system implemented widespread MRSA nasal PCR testing. All pharmacists were authorized to order testing and monitor results for patients receiving antimicrobial therapy for pneumonia or sepsis of suspected respiratory origin.

## Methods

This study evaluated the impact of MRSA nasal PCR initiation combined with active pharmacist ordering, monitoring, and intervention on the use of anti-MRSA agents. We used a multi-center, retrospective, pre-post cohort design across four community teaching hospitals (total 1,415 beds; facility range, 66–548 beds). All hospitals had established antimicrobial stewardship programs (ASPs) prior to the intervention, each combining preauthorization with postprescriptive audit and feedback. ASPs operated independently but followed largely harmonized guidelines and protocols.

Clinical pharmacists at each facility are embedded in multidisciplinary care teams managing vancomycin and other targeted antimicrobials. Protocols enabled all pharmacists to order MRSA nasal PCR testing for patients receiving antibiotics in cases where pneumonia or sepsis with suspected respiratory origin was a concern. Test results informed recommendations relayed to primary care teams. Pharmacist ordering and monitoring occurred seven days a week on day shifts.

MRSA PCR testing was introduced at three hospitals in early 2023 and at the fourth hospital in September 2023. Cohorts were compared between preintervention (January 2021–December 2022) and postintervention (January 2023–December 2024) periods, excluding 2023 data for hospital 4 due to later implementation. Anti-MRSA standardized antimicrobial administration ratio (SAAR) trends were also compared within the preintervention cohort (2021 vs 2022) to investigate prePCR testing implementation trends. This study complied with Strengthening the Reporting of Observational Studies in Epidemiology guidelines and was approved by the Trinity Health Ann Arbor Institutional Review Board.

Data was collected from National Healthcare Safety Network (NHSN) Antimicrobial Utilization and Resistance Module reporting and electronic medical record dashboards. Diagnoses were identified from International Classification of Diseases, 10th Revision, Clinical Modification (ICD-10-CM) coding.

Outcomes included changes in SAAR data and days of therapy (DOTs) per 1,000 days present, analyzed at the facility and patient care unit levels.^7^ NHSN-defined anti-MRSA agents included formulary antibiotics: intravenous vancomycin, linezolid, daptomycin, and ceftaroline. SAAR changes were assessed using the mid-P exact test (Poisson distribution), and categorical variables with Pearson’s χ^2^ test. Analyses used the NHSN statistics calculator and IBM SPSS Statistics v. 26.0 (IBM Corp., Armonk, NY, USA).^7^ Statistical significance was set at *P* ≤ .05.

## Results

Following implementation, 7,923 MRSA PCR tests were ordered for 7,608 unique encounters. Of these, 2,291 (28.9%) were positive for any *Staphylococcus* species and 791 (10%) were positive for MRSA, with MRSA positivity rates varying by hospital (8.4%–13.3%, *P* < .001).

Most tests (5,979 [75.5%]) were ordered for patients with suspected or confirmed respiratory infections and pharmacists ordered 1,974 (24.9%) of all tests. Pharmacists appropriately ordered PCR testing in cases with confirmed respiratory infections more frequently than non-pharmacists (1,868 of 1,974 [94.6%] vs 4,111 of 5,949 [69.1%]; *P* < .001).

**Figure 1.**
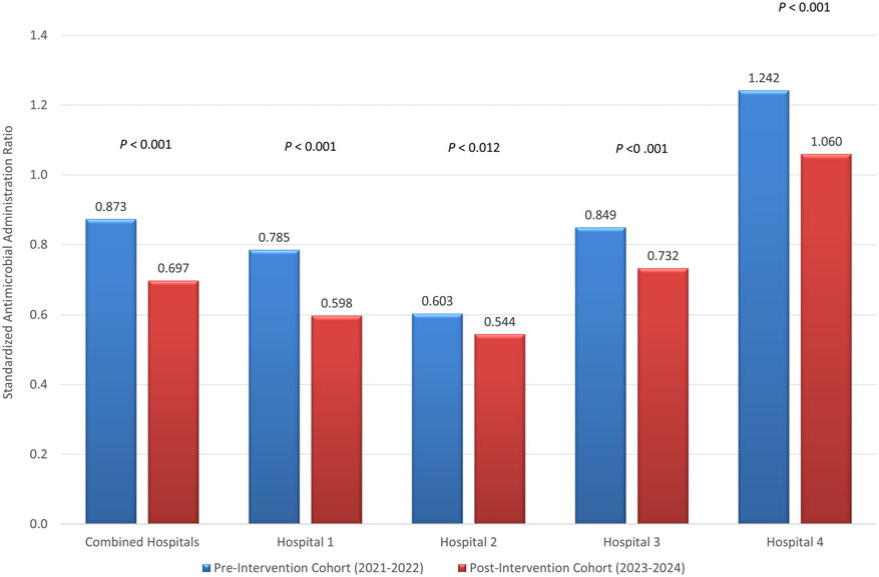
Anti-MRSA agent standardized antimicrobial administration ratios. Graph compares SAARs for Anti-MRSA agents between preintervention and postintervention cohorts.

Prior to testing implementation, anti-MRSA SAAR increased overall within the preintervention cohort between 2021 and 2022 (0.846 vs 0.877; *P* < .001). Postintervention anti-MRSA agent utilization significantly decreased with a 20.2% overall decrease in SAAR (0.873 vs 0.697; *P* < .001) (Figure 1). This resulted in 8,124 fewer DOTs than predicted use. All hospitals recorded significant SAAR decreases, ranging from 9.8% (*P* = .012) to 23.9% (*P* < .001).

**Figure 2.**
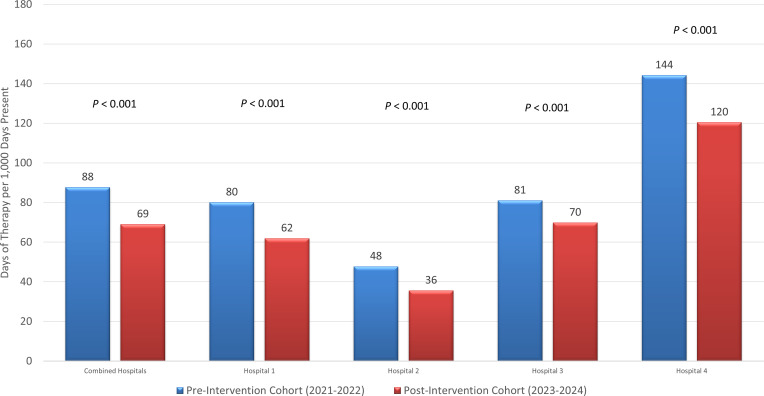
Anti-MRSA agent days of therapy per 1,000 days present. Graph compares anti-MRSA agent days of therapy per 1,000 days present between preintervention and postintervention cohorts.

DOTs per 1,000 days present also decreased 21.3% (88 DOTs per 1,000 days present vs 69 DOTs per 1,000 days present; *P* < .001) (Figure 2). Actual decreases by hospitals ranged from 11 to 24 DOTs per 1,000 days present. Vancomycin accounted for 91% and 87.4% of anti-MRSA agent utilization in each respective cohort.

Of 23 patient care units, 21 (91.3%) showed decreased utilization, and 17 (73.9%) exhibited significant SAAR decreases. Intensive care units had a 31.2% SAAR decrease (0.988 vs 0.68; *P* < .001); overall mean unit SAAR declined 20.3% from baseline (0.865 vs 0.689; *P* < .001).

## Discussion

Implementation of a MRSA nasal PCR testing with active pharmacist ordering, monitoring, and intervention significantly decreased the use of anti-MRSA antimicrobials across four community teaching hospitals. These results reinforce evidence that empowering pharmacists and incorporating MRSA nasal PCR screening into antimicrobial stewardship initiatives reduces unnecessary anti-MRSA therapy.^2,4–6^

A notable strength of this study is the multi-center design, spanning four different community teaching hospitals. Whereas prior research is often limited to single-center academic settings, this study’s approach allowed us to evaluate the impact across varied institutional contexts. Despite baseline differences in prescribing cultures, stewardship processes, and patient populations, all institutions demonstrated substantial reductions in anti-MRSA agent use, enhancing external validity.

Few studies have analyzed SAAR changes to evaluate ASP interventions. Pettit et al. reported reduced anti-MRSA agent SAARs after pharmacy-driven MRSA PCR implementation.^2^ Our study provides further evidence for using SAAR as a valuable antimicrobial stewardship metric.

Pharmacist-driven MRSA PCR ordering and monitoring may be especially valuable in settings with limited resources. Pharmacists in our study consistently ordered PCR testing appropriately, enhancing diagnostic stewardship. Enabling pharmacists to autonomously order MRSA PCR testing, especially when overseeing vancomycin dosing and therapeutic drug monitoring, expands the reach and impact of stewardship initiatives, underscoring their essential role in optimizing care. Integrating ordering and analysis within daily pharmacy workflows facilitates real-time intervention and highlights the vital role of all pharmacists in antimicrobial stewardship.^8^

Limitations include a single health-system setting, which may not capture variations across geographic regions, healthcare systems, or ASP and pharmacy practice models. Anti-MRSA agent utilization reflected all conditions, and not solely pneumonias to which pharmacist ordering was limited. However, no other significant antimicrobial stewardship initiatives targeted anti-MRSA agents during this period. A substantial portion of tests (2,069 [34.8%]) were ordered by non-pharmacists for non-respiratory infections. Daily prospective audit and feedback are standard for anti-MRSA agent orders. It’s likely that PCR tests for non-respiratory infections were evaluated by pharmacists as part of daily surveillance; however, guidelines non-respiratory interpretation were not outlined, and pharmacist recommendations likely varied. The impact of testing for non-respiratory infections remains unclear. Additionally, we did not collect detailed clinical outcome data, adjudicate endpoints, or formally analyze costs.

In summary, MRSA nasal PCR testing introduction with pharmacist ordering, monitoring, and intervention significantly decreased anti-MRSA agent utilization in a multi-hospital health-system. Empowering pharmacists’ autonomy in this stewardship role represents an effective, scalable strategy to optimize antimicrobial use across diverse hospital settings.
